# Are YouTube Videos on the Treatment of Macular Holes Useful for Patients?

**DOI:** 10.7759/cureus.34767

**Published:** 2023-02-08

**Authors:** Senol Sabanci, Muhammet Kazim Erol, Birumut Gedik, Lütfiye Yaprak, Elcin Suren

**Affiliations:** 1 Ophthalmology, Antalya Training and Research Hospital, Antalya, TUR; 2 Ophthalmology, Antalya Serik State Hospital, Antalya, TUR

**Keywords:** macular hole, youtube, video, jama, discern

## Abstract

Objective

In this study, we aimed to assess the effectiveness and quality of the YouTube videos for patients regarding the treatment of macular holes.

Materials and methods

The quality and effectiveness of the videos on macular hole treatment on YouTube were evaluated using the DISCERN, global quality score (GQS), Journal of the American Medical Association (JAMA), and usefulness index scoring systems.

Results

The median DISCERN score of 100 videos included in the study was 18 (range: 15-73), the median JAMA score was 3 (range: 0-4), the median global quality score was 1 (range: 1-5), and the median usefulness index score was 1 (range: 0-5). The JAMA scores of the videos uploaded by non-clinicians were found to be significantly lower than those of the videos uploaded by clinicians (p<0.001). However, the daily viewing rate and the number of comments and likes for the videos uploaded by non-clinicians were found to be significantly higher than those for the videos uploaded by clinicians (p<0.003).

Conclusion

Based on our findings, while all of the beneficial macular hole videos on YouTube were published by clinicians, the majority of them are not really helpful to patients.

## Introduction

A macular hole is a retinal disorder that is usually seen in people of advanced ages and it affects the central vision. Even though the majority of them are largely idiopathic, secondary causes can occasionally be involved [1]. Although the treatment method for a macular hole is determined by its degree, a vitrectomy is typically employed. Other therapeutic options include intravitreal injection of medications such as ocriplasmin. With various surgical techniques devised in the last several years, vitrectomy has seen a big improvement in functional success and a large improvement in anatomical success [2]. The internet has become a major source of medical information in recent years, and nearly all patients use the internet to research their complaints and illnesses. YouTube is among the most well-known of these sites. Of note, 79% of internet users have a YouTube account, and with 2.3 billion monthly active users, over one billion hours of video viewing, and over 720,000 hours of video posting each day, the rate at which YouTube is used has risen astronomically over the past few years [3]. Also, YouTube has established itself as a very popular source of health-related information. However, the ease with which videos may be uploaded to YouTube and the fact that anybody, expert or not, is able to do so raise concerns about the validity of the material and the potential for spreading misinformation. As a response to these concerns, studies that assess the validity and substance of health-related videos posted on social media platforms have started to appear in recent years [4]. In this study, we assessed the YouTube videos regarding macular hole therapy for their quality, dependability, and level of benefit to patients.

## Materials and methods

On December 22, 2021, the first 120 videos were seen after signing out of the YouTube site and typing in "macular hole therapy for patients." The study included videos in English that are more than a minute long. Videos that were duplicated, unrelated to macular holes, without audio or subtitles, less than one minute in length, not in English, or those in which comments had been deleted by the uploader were excluded from our study. The number of times the video has been viewed, its length, its age (from the time it was uploaded until January 22, 2022), the number of likes and comments it has received, who uploaded it (a patient, a healthcare professional, a hospital or academic institution, a commercial health channel), its purpose (clinical information, treatment, and postoperative period), and its content (scientific, patient information, patient experience), were all evaluated in the study. The utility level of the videos was also evaluated by using the DISCERN, global quality score (GQS), Journal of the American Medical Association (JAMA), and usefulness index scoring systems (Tables 1-4).

**Table 1:** DISCERN scoring system

Section 1	What is investigated? Is the publication reliable?	No	Partially	Yes
1	Are the aims clear?	1	2-3	4-5
2	Does it achieve its aims?	1	2-3	4-5
3	Is it relevant?	1	2-3	4-5
4	Is it clear what sources of information were used to compile the publication (other than the author or producer)?	1	2-3	4-5
5	Is it clear when the information used or reported in the publication was produced?	1	2-3	4-5
6	Is it balanced and unbiased?	1	2-3	4-5
7	Does it provide details of additional sources of support and information?	1	2-3	4-5
8	Does it refer to areas of uncertainty?	1	2-3	4-5
Section 2	How good is the quality of information regarding treatment choices?			
9	Does it describe how each treatment works?	1	2-3	4-5
10	Does it describe the benefits of each treatment?	1	2-3	4-5
11	Does it describe the risks of each treatment?	1	2-3	4-5
12	Does it describe what would happen if no treatment is used?	1	2-3	4-5
13	Does it describe how the treatment choices affect the overall quality of life?	1	2-3	4-5
14	Is it clear that there may be more than 1 possible treatment choice?	1	2-3	4-5
15	Does it provide support for shared decision-making?	1	2-3	4-5
Section 3	Overall rating of the publication			
16	Based on the answers to all of these questions, rate the overall quality of the publication as a source of information about treatment choices	1	2-3	4-5
		1: Low	2-3: Moderate	4-5: High

**Table 2:** Usefulness score criteria

Usefulness score		
Score criteria	Not mentioned	Mentioned
Definition	0	1
Indication	0	1
Procedure involved	0	1
Complication	0	1
Prognosis-survival	0	1

**Table 3:** JAMA score criteria JAMA: Journal of the American Medical Association

JAMA score		
Authorship: authors and contributors, their affiliations, and relevant credentials should be provided (0 or 1 point)		
Attribution: references and sources for all content should be listed clearly, and all relevant copyright information should be noted (0 or 1 point)		
Disclosure: website “ownership” should be prominently and fully disclosed, as should any sponsorship, advertising, underwriting, commercial funding arrangements or support, or potential conflicts of interest (0 or 1 point)		
Currency: dates when content was posted and updated should be indicated (0 or 1 point)		

**Table 4:** Global quality scoring system

Global quality score		
1 - Poor quality, very unlikely to be of any use to patients		
2 - Poor quality but some information present, of very limited use to patients		
3 - Suboptimal flow, some information covered but important topics missing, somewhat useful to patients		
4 - Good quality and flow, most important topics covered, useful to patients		
5 - Excellent quality and flow, highly useful to patients		

The DISCERN system, which has three parts and 16 questions that are scored on a scale of 1 to 5, is used to assess the content, balance, and clarity of each given publication's arguments. The first eight questions focus on dependability, the next seven provide treatment information, and the last question assesses overall quality. Scores for the videos range from 16 to 75, categorized as follows: 63-75: excellent, 51-62: good, 39-50: fair, 27-38: poor, and 16-26: extremely poor [5].

Another rating system, JAMA, rates the validity of online health resources on a scale of 0 to 1, taking into consideration the authors' clarity, references and citations, declaration of any financial or other relationships with commercial interests, timeliness, and the date the data was posted [6]. Another grading method, the global quality score, gives a score between 1 and 5 depending on the uploaded video's content, quality, and chance of helping patients. Another rating method is the video usefulness index, which gives points to materials based on the disease diagnosis, treatment indication, technique, prognosis, and consequences. Each item receives 1 point in this scoring system, which is then summed up. Scores of 0 and 1 are seen as having limited value for patients since they provide little information and video material. A score of 2-3 shows that the information and substance are of decent quality, but the patients are still not sufficiently educated. A score of 4-5 indicates that the content is sufficiently capable of educating patients and the information is of excellent quality and substance [7].

Statistical analysis

For descriptive statistics, mean ± standard deviation (SD) was used to present continuous data with normal distribution. The median with minimum-maximum values was applied for continuous variables without normal distribution. Numbers and percentages were used for categorical variables. The Shapiro-Wilk, Kolmogorov-Smirnov, and Anderson-Darling tests were used to analyze the normal distribution of the numerical variables. The Mann-Whitney U test was employed to compare two independent groups where numerical variables had no normal distribution. The Kruskal-Wallis H test was used to compare more than two independent groups where numerical variables had no normal distribution. The Dwass-Steel-Critchlow-Fligner test was utilized to evaluate differences between the groups if the non-parametric tests were applied. The Pearson Chi-Square and Fisher's exact tests were used to compare the differences between categorical variables in 2x2 tables. The Fisher-Freeman Halton test was used in RxC tables. Spearman correlation coefficients were calculated to analyze the relationships between the videos’ descriptive statistics and characteristics. Jamovi (Version 2.2.5.0) and JASP (Version 0.16.1) were used for statistical analysis. A p-value of 0.05 was considered statistically significant.

## Results

Of the 120 videos we watched, 100 were included in the study; nine videos unrelated to the macular hole, four videos without sound, three duplicated videos, two videos shorter than one minute, and two non-English videos were excluded from the study. Descriptive statistics are presented in Table 5. The median length of the videos was 4.2 minutes, ranging from one to 136.2 minutes. The majority (60.0%) were less than five minutes in length. They received a median of 1889.5 views with a viewing rate of 1.6 per day. The median number of “likes” was 16, ranging from 0 to 491.

**Table 5:** Data on the length, number of views, comments, and likes related to the videos

Variables	Values (n=100)
Video length, minutes, median (range)	4.2 (1–136.2)
Groups by video length, n (%)	
<5 minutes	60 (60)
5-10 minutes	22 (22)
≥10 minutes	18 (18)
Days since upload, median (range)	821 (30–5091)
Number of views, median (range)	1189.5 (13–95938)
Daily viewing rate, median (range)	1.6 (0–54.8)
Number of comments, median (range)	2 (0–272)
Number of likes, median (range)	16 (0–491)

Video characteristics are shown in Table 6. Healthcare professionals uploaded the majority of the videos (93%). Grouping based on the upload source revealed that healthcare professionals (40%), commercial health channels (28%), and hospital or academic institutions (25%) were the most frequent sources of the videos. The purpose of the videos was related to clinical information and treatment in most cases (Table 6). More than half of the videos (63%) had a scientific context. The calculations regarding the total scores of the different quality metrics are detailed in Table 6. Regarding the usefulness score, more than half of the videos (67%) scored 0-1. 

**Table 6:** Data on the authorship, upload source, purpose, and content of the videos JAMA: Journal of the American Medical Association

Variables	Values (n=100)
Personal source of authorship, n (%)	
Healthcare professional	93 (93)
Non-healthcare individual users	7 (7)
Upload source, n (%)	
Healthcare professional	40 (40)
Hospital/academic institution	25 (25)
Commercial health channel	28 (28)
Patient	7 (7)
Purpose, n (%)	
Clinical information	8 (8)
Treatment	62 (62)
Clinical information and treatment	26 (26)
Postoperative period	4 (4)
Content, n (%)	
Scientific	63 (63)
Patient information	29 (29)
Patient experience	8 (8)
DISCERN score, median (range)	18 (15–73)
JAMA score, median (range)	3 (1–4)
Global quality score, median (range)	1 (1–5)
Usefulness score, median (range)	1 (0–5)
Usefulness score categories, n (%)	
0-1	67 (67)
2-3	21 (21)
4-5	12 (12)

There were significant differences between the descriptive statistics and characteristics of the videos uploaded by healthcare professionals and those of the videos uploaded by non-healthcare users (Table 7). The videos uploaded by non-healthcare individual users were significantly longer (p=0.002), had more views (p=0.003), comments (p<0.001), and “likes (p<0.001), and had a higher rate of daily viewing (p=0.001).

**Table 7:** Comparison of the data on videos based on the source of authorship *Mann-Whitney U test; **Pearson Chi-square or Fisher-Freeman-Halton test JAMA: Journal of the American Medical Association

Variables	Source of authorship		P-value
	Healthcare professionals (n=93)	Non-healthcare users (n=7)	
Video length, minutes, median (range)	4.0 (1.0–136.2)	13.1 (5.2–16.4)	0.002*
Groups by video length, n (%)			
<5 minutes	60 (64.5)	0 (0)	<0.001**
5-10 minutes	21 (22.6)	1 (14.3)	
≥10 minutes	12 (12.9)	6 (85.7)	
Days since upload, median (range)	821 (30–5091)	790 (274–1701)	0.766*
Number of views, median (range)	969 (13–95938)	7514 (2061–29775)	0.003*
Daily viewing rate, median (range)	1.2 (0–54.8)	12.6 (2.6–34.1)	0.001*
Number of comments, median (range)	1 (0–254)	54 (11–272)	<0.001*
Number of likes, median (range)	14 (0–491)	130 (50–419)	<0.001*
Upload source, n (%)			
Healthcare professional	40 (43)	0 (0)	<0.001**
Hospital or academic institution	25 (26.9)	0 (0)	
Commercial health channel	28 (30.1)	0 (0)	
Patient	0 (0)	7 (100)	
Purpose, n (%)			
Clinical information	6 (6.5)	2 (28.6)	0.038**
Treatment	60 (64.5)	2 (28.6)	
Clinical information and treatment	24 (25.8)	2 (28.6)	
Postoperative period	3 (3.2)	1 (14.3)	
Content, n (%)			
Scientific	63 (67.7)	0 (0)	<0.001**
Patient information	29 (31.2)	0 (0)	
Patient experience	1 (1.1)	7 (100)	
DISCERN score, median (range)	17 (15–73)	21 (16–29)	0.458*
JAMA score, median (range)	3 (1–4)	2 (1–2)	<0.001*
Global quality score, median (range)	1 (1–5)	2 (1–3)	0.138*
Usefulness score, median (range)	1 (0–5)	2 (0–3)	0.698*
Usefulness score categories, n (%)			
0-1	64 (68.8)	3 (42.9)	0.074**
2-3	17 (18.3)	4 (57.1)	
4-5	12 (12.9)	0 (0)	

We detected significant differences regarding the upload source, purpose, and content of the videos (p<0.05) (Table 7). The scores of the quality metrics (DISCERN, global quality score, and usefulness score) were similar between the groups (p=0.458, p=0.138, p=0.698, respectively) (Table 7). The videos uploaded by non-healthcare users had a lower median JAMA score than those by healthcare professionals (p<0.001).

Table 8 presents a comparative analysis of the descriptive statistics and characteristics of the videos according to the upload sources. There were significant differences between the groups (p<0.05). The length of the videos uploaded by patients was significantly longer than those by healthcare professionals (p=0.033), hospital or academic institutions (p=0.008), and healthcare channels (p=0.014). We detected significant differences in the number of views and daily view rates between the videos by patients and those by healthcare professionals (p=0.026 and p=0.002, respectively) and healthcare channels (p=0.016 and p=0.009, respectively). The viewing rate per day was significantly higher for videos by patients than those uploaded by hospitals or academic institutions (p=0.049). The number of comments and “likes” for the videos uploaded by patients was significantly higher than for those uploaded by healthcare professionals (p<0.001 and p=0.001, respectively), hospitals/academic institutions (p<0.001 and p=0.005, respectively), and healthcare channels (p<0.001 and p<0.001, respectively).

**Table 8:** Comparison of the data on videos based on the upload source *Kruskal-Wallis H test; **Pearson Chi-square or Fisher-Freeman-Halton test JAMA: Journal of the American Medical Association

Variables	Upload source				P-value
	Healthcare professionals (n=40)	Hospitals/academic institutions (n=25)	Healthcare channels (n=28)	Patients (n=7)	
Video length, minutes, median (range)	4.8 (1–136.2)	3.2 (1–31.2)	3.1 (1–58)	13.1 (5.2–16.4)	0.005*
Groups by video length, n (%)					
<5 minutes	20 (50)	19 (76)	21 (75)	0 (0)	<0.001**
5-10 minutes	15 (37.5)	3 (12)	3 (10.7)	1 (14.3)	
≥10 minutes	5 (12.5)	3 (12)	4 (14.3)	6 (85.7)	
Days since upload, median (range)	896.5 (145–5091)	761 (30–3440)	852 (31–4352)	790 (274–1701)	0.732*
Number of views, median (range)	777 (13–82138)	1214 (25–64531)	876 (42–95938)	7514 (2061–29775)	0.024*
Daily viewing rate, median (range)	1.1 (0–19.3)	1.7 (0.1–54.8)	1 (0–25.2)	12.6 (2.6–34.1)	0.004*
Number of comments, median (range)	2.5 (0–254)	0 (0–30)	1 (0–65)	54 (11–272)	<0.001*
Number of likes, median (range)	14.5 (0–168)	16 (0–491)	10.5 (0–78)	130 (50–419)	0.001*
Source of authorship, n (%)					
Healthcare professionals	40 (100)	25 (100)	28 (100)	0 (0)	<0.001**
Non-healthcare individual users	0 (0)	0 (0)	0 (0)	7 (100)	
Purpose, n (%)					
Clinical information	0 (0)	4 (16)	2 (7.1)	2 (28.6)	<0.001**
Treatment	33 (82.5)	9 (36)	18 (64.3)	2 (28.6)	
Clinical information and treatment	7 (17.5)	12 (48)	5 (17.9)	2 (28.6)	
Postoperative period	0 (0)	0 (0)	3 (10.7)	1 (14.3)	
Content, n (%)					
Scientific	34 (85)	9 (36)	20 (71.4)	0 (0)	<0.001**
Patient information	6 (15)	15 (60)	8 (28.6)	0 (0)	
Patient experience	0 (0)	1 (4)	0 (0)	7 (100)	
DISCERN score, median (range)	16 (15–69)	29 (16–64)	17 (16–73)	21 (16–29)	0.004*
JAMA score, median (range)	3 (1–4)	3 (1–3)	3 (1–4)	2 (1–2)	0.004*
Global quality score, median (range)	1 (1–4)	3 (1–5)	1 (1–4)	2 (1–3)	0.001*
Usefulness score, median (range)	1 (0–5)	1 (0–5)	1 (0–4)	2 (0–3)	0.165*
Usefulness score categories, n (%)					
0-1	29 (72.5)	13 (52)	22 (78.6)	3 (42.9)	0.048**
2-3	8 (20)	5 (20)	4 (14.3)	4 (57.1)	
4-5	3 (7.5)	7 (28)	2 (7.1)	0 (0)	

The DISCERN score for the videos uploaded by hospitals or academic institutions was significantly higher than for those uploaded by healthcare professionals (p=0.007) and healthcare channels (p=0.049). JAMA scores for videos by patients were significantly lower than for videos by healthcare professionals (p=0.002), hospitals or academic institutions (p=0.015), and healthcare channels (p=0.024). Global quality scores for videos uploaded by hospitals or academic institutions were significantly higher than for those by healthcare professionals (p=0.007) and healthcare channels (p=0.017). Although the median value of the usefulness score was similar between the groups (p=0.165), the categories based on this score revealed significant differences (p=0.048) (Table 8).

Although the median length of the videos according to the usefulness score categories was similar (p=0.321), we detected significant differences between the categories regarding the duration of the videos and the usefulness score categories (p=0.001). The descriptive statistics were similar between the usefulness score categories (p>0.05) (Table 9).

**Table 9:** Comparison of the data on videos based on usefulness score categories *Kruskal-Wallis H test; **Pearson Chi-square or Fisher-Freeman-Halton test JAMA: Journal of the American Medical Association

Variables	Usefulness score categories			P-value
	0-1 (n=67)	2-3 (n=21)	4-5 (n=12)	
Video length, minutes, median (range)	4 (1–58)	4.3 (1–136.2)	4.3 (1.4–56.2)	0.321*
Groups by video length, n (%)				
<5 minutes	42 (62.7)	11 (52.4)	7 (58.3)	0.001**
5-10 minutes	20 (29.9)	1 (4.8)	1 (8.3)	
≥10 minutes	5 (7.5)	9 (42.9)	4 (33.3)	
Days since upload, median (range)	897 (31–5091)	641 (152–3011)	716.5 (30–2861)	0.114*
Number of views, median (range)	1574.0 (13–95938)	491 (39–21458)	720.5 (25–64531)	0.233*
Daily viewing rate, median (range)	1.7 (0.1–54.8)	0.9 (0–34.1)	2 (0.1–32)	0.448*
Number of comments, median (range)	2 (0–254)	0 (0–272)	0.5 (0–30)	0.608*
Number of likes, median (range)	18 (0–158)	8 (0–419)	8 (1–491)	0.349*
Purpose, n (%)				
Clinical information	7 (10.4)	1 (4.8)	0 (0)	<0.001**
Treatment	50 (74.6)	11 (52.4)	1 (8.3)	
Clinical information and treatment	7 (10.4)	8 (38.1)	11 (91.7)	
Postoperative period	3 (4.5)	1 (4.8)	0 (0)	
Content, n (%)				
Scientific	49 (73.1)	9 (42.9)	5 (41.7)	0.009**
Patient information	14 (20.9)	8 (38.1)	7 (58.3)	
Patient experience	4 (6)	4 (19)	0 (0)	
DISCERN score, median (range)	16 (15–32)	29 (16–53)	47.5 (30–73)	<0.001*
JAMA score, median (range)	3 (1–4)	3 (1–4)	3.0 (2–4)	0.697*
Global quality score, median (range)	1 (1 –3)	3 (1–4)	4.0 (1–5)	<0.001*

There were significant differences in the distribution of the categories based on the purpose and content of the videos between the groups (p<0.05) (Table 9). The comparison of the JAMA scores revealed no significant differences (p=0.697). However, the videos with a usefulness score of 4-5 had significantly higher values of the DISCERN and global quality score than those with a score of 2-3 (p<0.001 and p=0.001, respectively) and a score of 0-1 (p<0,001 and p<0.001, respectively). We detected significant differences in the values of the DISCERN and global quality scores between the usefulness scores of 2-3 and 0-1 (p<0.001 and p<0.001, respectively) (Table 9).

The results of the correlation analysis of the descriptive statistics and video characteristics are detailed in Table 10. The DISCERN score positively correlated with the global quality score (r=0.796, p<0.001) and the usefulness score (r=0.823, p<0.001). There was a positive correlation between the global quality score and the usefulness score (r=0.690, p<0.001).

**Table 10:** Data on correlations between various aspects of the videos Spearman's rho correlation coefficient JAMA: Journal of the American Medical Association

Variables		Video length, minutes	Days since upload	Number of views	Daily viewing rate	Number of comments	Number of likes	DISCERN score	JAMA score	Global quality score
Days since upload	r	0.000	—							
	P-value	0.994	—							
Number of views	r	0.138	0.555	—						
	P-value	0.171	<0.001	—						
Daily viewing rate	r	0.180	0.078	0.845	—					
	P-value	0.074	0.440	<0.001	—					
Number of comments	r	0.221	0.139	0.582	0.610	—				
	P-value	0.027	0.169	<0.001	<0.001	—				
Number of likes	r	0.167	0.179	0.774	0.801	0.712	—			
	P-value	0.097	0.076	<0.001	<0.001	<0.001	—			
DISCERN score	r	0.102	-0.214	-0.061	0.056	-0.215	-0.112	—		
	P-value	0.311	0.033	0.544	0.581	0.031	0.267	—		
JAMA score	r	-0.077	-0.081	-0.272	-0.238	-0.327	-0.299	0.109	—	
	P-value	0.444	0.422	0.006	0.017	<0.001	0.002	0.279	—	
Global quality score	r	-0.088	-0.125	0.001	0.085	-0.101	-0.056	0.796	-0.008	—
	P-value	0.382	0.214	0.990	0.400	0.320	0.583	<0.001	0.938	—
Usefulness score	r	0.148	-0.221	-0.125	-0.009	-0.142	-0.172	0.823	0.149	0.690
	P-value	0.141	0.027	0.216	0.930	0.157	0.086	<0.001	0.139	<0.001

## Discussion

Today, the internet has become an almost indispensable tool in our everyday lives. The internet, which we utilize to research any topic under the sun, is commonly used for medicine-related search/research as well. One of the social media sites, YouTube, is now the second-most-used search resource after Google [8]. The fact that YouTube is widely utilized, the ease with which to submit videos on it, and that it is free have made it a gathering place for both people seeking knowledge and those wanting to share it. All of these carry some dangers as well as potential benefits. One of these risks would be that people could be given inaccurate or incomplete information by others who lack appropriate knowledge or who have vested personal or professional interests. According to Şahin et al., one-third of the YouTube videos on retinopathy of prematurity were erroneous and could have negative effects [9]. Owing to these factors, some people might decide not to get certain therapies, while others may demand unrealistically high results. For the reasons explained above, there has been an increase in recent years in the studies on the validity, impartiality, content, and quality of health details available on YouTube [10].

Our review of the literature revealed scarce research analyzing the caliber, substance, and dependability of YouTube videos related to macular holes. We reviewed YouTube videos regarding treatment for macular holes for the purpose of this study. Relevant videos were graded in this study based on the DISCERN, JAMA, global quality score, and usefulness scoring index. We had to use the median value rather than the mean value since vastly different values were obtained within the results of each scoring system. The median scores for DISCERN, JAMA, and usefulness and global quality scores were assessed to be 18, 3, 1, and 1, respectively, in this study. Clinical videos submitted by nonphysicians had an average JAMA score significantly lower than clinical videos uploaded by physicians. The JAMA score offers important details on the dependability of the submitted video as it assesses the video's origin, the person who shot it, any possible sponsors or financial ties, the video's relevance, and references [6]. Therefore, their high JAMA scores lead us to conclude that clinical videos uploaded are more trustworthy. Between clinicians and non-clinicians, the mean scores related to other scoring systems were not significant (global quality score, usefulness index, DISCERN) [9,11]. The median DISCERN score in this study was 18, which was shown to be far lower than in similar investigations on other individuals. We may infer that this is because most videos regarding macular holes are scientific in nature and only discuss surgery, skipping over symptoms, causes, types of therapy, side effects, and prognosis. This disease is less widespread than other illnesses, which means less commercial return, as well as a low number of physicians interested in this issue. Due to these reasons, macular hole videos may have been uploaded mostly for scientific purposes rather than informing patients. 

The content and purpose of the videos produced by healthcare professionals and those uploaded by non-healthcare professionals were found to be significantly different (p<0.05). The highest DISCERN score among the videos uploaded by non-clinicians was 29. This was due to the fact that patients frequently recorded videos explaining why they underwent surgery and the recommended head posture following surgery. Only three of the videos analyzed were found to have DISCERN scores of 63 or above and very high content standards, and these were all submitted by physicians. Only five videos in the review met all the usefulness score criteria (indication, description, surgical technique, complication, and prognosis), and all of these videos were posted by clinicians. Based on our analysis of who uploaded the videos reviewed in other studies, we observe that there is a difference between clinicians and non-clinicians, but this difference is more pronounced in our study [5,12,13]. This could be attributed to the fact that the macular hole is less common than the other reviewed subjects and subsequently less popular. Another explanation could be that macular holes are typically diagnosed in elderly patients, who are also less likely to utilize social media to describe their experiences. In this study, it was shown that videos submitted by non-clinicians had more views and a higher daily view rate (Table 7). We may explain this by pointing out that since they do not use medical terminology, persons with the same ailment tend to be more inquisitive about what others are going through. The fact that surgical videos for scientific purposes make up the majority of the videos produced by physicians regarding macular holes, and that some of them contain lengthy videos in the form of seminars, might be a factor in the dearth of views. Based on an analysis of the number of comments and likes, it was found that the videos uploaded by non-clinicians obtained more comments and likes than the videos uploaded by clinicians (p<0.001). This is explained by the fact that videos with more viewership receive more likes and comments. Since YouTube hides the number of dislikes, it was not possible to calculate the number of dislikes or the viewer interaction index in this study.

Based on the above-mentioned findings, there are several videos concerning macular holes on YouTube that have been posted by medical professionals, healthcare organizations (hospitals and academic institutions), commercial health channels, and patients. Most of these videos were produced for scientific purposes, and few of them are actually helpful to patients. It has been shown that even the videos uploaded by professionals do not go into adequate detail regarding available treatments, side effects, and prognosis. Most videos released in recent years have been about treating macular holes. It has been observed that some videos on advancements in science and surgery have been produced. There is a remarkable number of videos discussing the inverted flap procedure, which is used to repair big and refractory macular holes. It appears that individuals will increasingly use YouTube in the coming years to obtain knowledge on health and healthcare, just as they do with every other subject.

## Conclusions

This is the first study to analyze the caliber, substance, and dependability of YouTube videos related to macular holes in the literature. Although YouTube videos could be incredibly beneficial for many, they could also have many negative effects. The quality of videos released on sensitive and significant subjects like healthcare is crucial. Clinicians should properly describe the disease symptoms, when the treatment will begin, any potential complications, and the potential benefits of the therapy in the video recordings they publish to educate the public without regard for profit. In order to inform people more thoroughly and objectively, it will be beneficial for specialists to rigorously review health-related videos before they are uploaded to YouTube.
